# Promises and Pitfalls in the Use of PD-1/PD-L1 Inhibitors in Multiple Myeloma

**DOI:** 10.3389/fimmu.2018.02749

**Published:** 2018-11-27

**Authors:** Stefania Oliva, Rossella Troia, Mattia D'Agostino, Mario Boccadoro, Francesca Gay

**Affiliations:** Myeloma Unit, Division of Hematology, University of Torino, Azienda Ospedaliero-Universitaria Città della Salute e della Scienza di Torino, Torino, Italy

**Keywords:** multiple myeloma, PD-1, PD-L1, immune dysregulation, T cells

## Abstract

In the biology of multiple myeloma (MM), immune dysregulation has emerged as a critical component for novel therapeutic strategies. This dysfunction is due to a reduced antigen presentation, a reduced effector cell ability and a loss of reactive T cells against myeloma, together with a bone marrow microenvironment that favors immune escape. The Programmed Death-1 (PD-1) pathway is associated with the regulation of T cell activation and with the apoptotic pathways of effector memory T cells. Specifically, the binding with PD-1 ligand (PD-L1) on the surface of tumor plasma cells down-regulates T cell-proliferation, thus contributing to the immune escape of tumor cells. In relapsed and/or refractory MM (RRMM) patients, PD-1/PD-L1 blockade was analyzed by using nivolumab, pembrolizumab, and durvalumab. Outcomes with single agents were unsatisfactory, whereas combination strategies with backbone immunomodulatory drugs (IMiDs) suggested a synergistic action in such a complex immunological landscape, even in patients previously refractory to these drugs. Nevertheless, these combinations were also associated with an increased incidence of adverse events. This review aims to analyze the available preclinical and clinical data on the role of PD-1/PD-L1 inhibitors in MM therapy, focusing on available preliminary efficacy and safety data and offering insights for future investigation.

## Introduction

In the pathogenesis of multiple myeloma (MM), the immortalization of a MM propagating cell is induced by an initiating “hit.” The subsequent accumulation of genetic “hits” in a multistep process leads to the typical MM characteristics: the proliferation of monoclonal plasma cells and the consequent overproduction of immunoglobulin or light chains that can cause end-organ damage and specific symptoms (i.e., bone disease, anemia, renal failure, and hypercalcemia) (1, 2). Moreover, an important role is also played by the interactions between the microenvironment—which includes the immune system where the tumor grows—and the MM cells (3). In general, the immune system can potentially recognize a tumor and reject it. Natural killer (NK) cells may detect tumor cells by their typical, although aspecific, tumor characteristics (such as upregulated cell stress ligands and/or downregulated major histocompatibility complex [MHC]) and kill them. Then, dendritic cells (DCs) and macrophages can internalize and process cell products and present derived molecules to B and T cells (4–6). T- and B-cell activation causes the proliferation of cell clones and the production of tumor-specific antibodies, with the final goals of eliminating the remaining tumor cells and generating immune memory to prevent tumor recurrence (7). Through this process, a strongly immunogenic tumor in a highly immunocompetent subject could potentially eradicate the tumor. In the cases of less immunogenic tumors and/or less immunocompetent individuals, some cancer cells can survive despite remaining under immunosurveillance. Nevertheless, at a certain point, changes in the tumor expression of antigens can allow the tumor to avoid immunosurveillance. Similarly, a weakened immune system can be less efficient in maintaining the tumor under control and, as a consequence, it favors the tumor escape (8). A progressive immune dysregulation strongly characterizes MM, whose plasma cells can easily escape immunosurveillance through many possible mechanisms, such as the deficient B-cell immunity, the expansion of regulatory T cells (Tregs), the DC dysfunction, and the reduction of T-cell cytotoxicity.

The potential role of immunosurveillance on tumor control is the rationale for the use of the immuno-oncology approach in cancer treatment, including MM. Immune checkpoint interactions have emerged as a major mechanism for immunosurveillance and evasion. Immune checkpoint blockade enhances antitumor immunity by blocking cytotoxic T-lymphocyte antigen 4 (CTLA-4) and programmed cell death 1 (PD-1) or PD-1 ligand (PD-L1). Monoclonal antibodies (mAbs) targeting checkpoint pathway on immune and tumor cells (known as checkpoint inhibitors) proved to be effective in several tumors. Ipilimumab, pembrolizumab, nivolumab, atezolizumab, durvalumab, and avelumab are currently approved by the Food & Drug Administration (FDA) (9). Check-point inhibitors also showed specific side effects, defined as immune-related AEs (irAEs): in fact, they can cause inflammation due to an increased activity of the immune system (9) (see section Immune-Related AEs). Results on solid tumors and on other hematologic cancers provided the basis to evaluate their effectiveness and safety in MM.

## Rationale for checkpoint inhibition in multiple myeloma

### PD-1/PD-L1 pathway in normal cells and myeloma cells

The immune dysfunction is critical for the genesis of MM and various cells are involved. NK cells show quantitative and functional changes, with a decrease during the advanced disease phase. In this sense, NK cell-mediated cytotoxicity (particularly when enhanced) is a promising target for immunotherapies, mainly for immunomodulatory drugs (IMiDs) and novel mAbs. Also T-cell immunity and the antigen-presenting ability of DCs present some issues: there is a selective loss of myeloma-specific lymphocytes (NKT-cells, γδ T cells) and a coexistent rise in suppressor cells, including regulatory T cells and MDSCs, within the bone marrow microenvironment and in the peripheral blood (10, 11).

**Figure 1:**
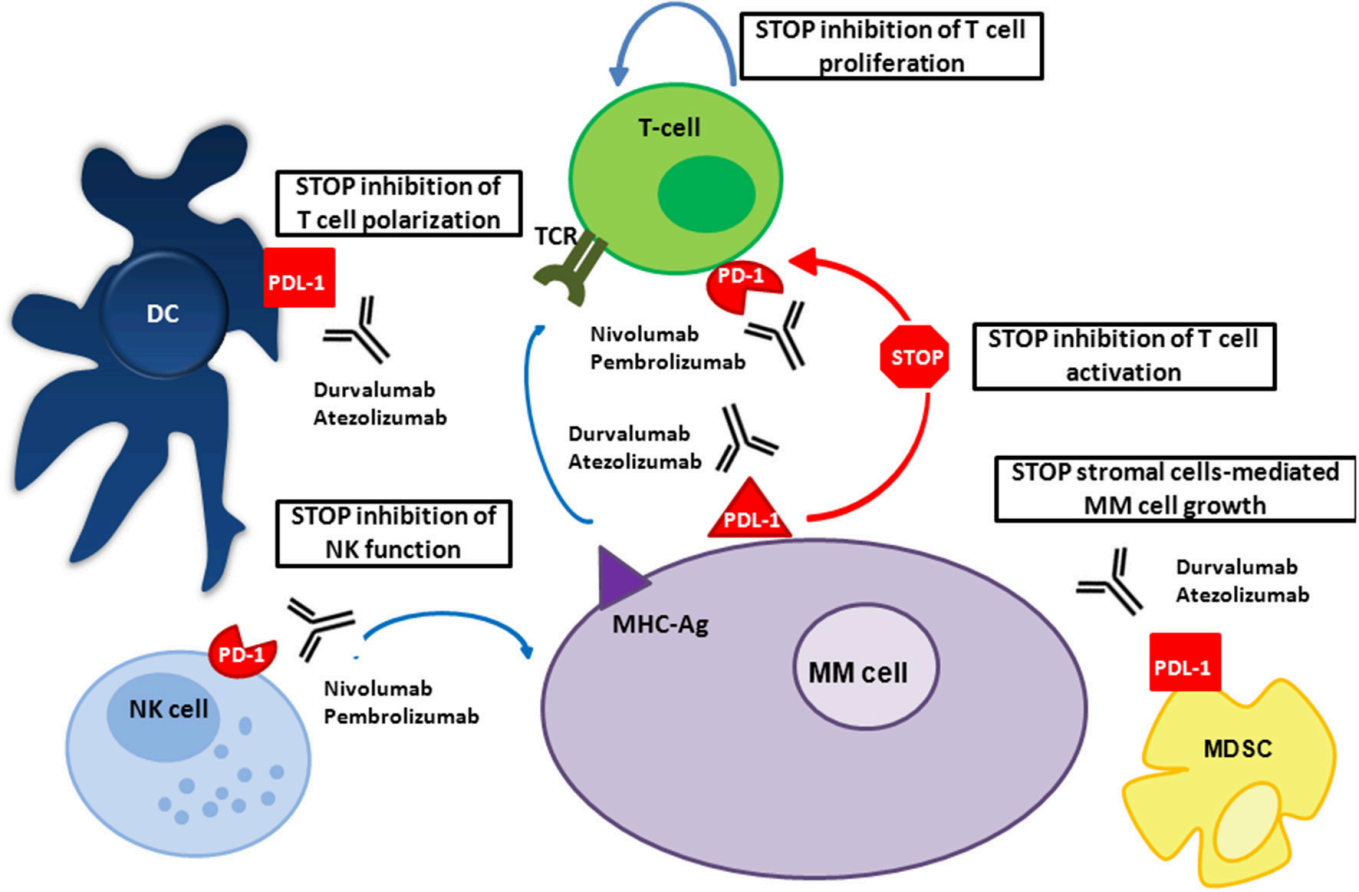
Mechanism of action of PD-1/PD-L1 inhibitors in MM. In patients with MM, PD-L1 is expressed on MM and bone marrow microenvironment accessory cells; PD-1 on NK cells and T cells. PD-1/PD-L1 signaling in patients with MM inhibits the function of these immune cells, allowing MM to escape death. Both anti-PD-1 and anti-PD-L1 mAbs prevent this interaction.

In the presence of malignant plasma cells, immune tolerance is fostered by immune checkpoint pathways, which usually help maintain the immune equilibrium. The PD-1 is part of the CD28 receptor family, and is expressed on activated B cells, monocytes, T cells, and NK T cells (12). PD-L1 and PD-L2 are expressed on antigen-presenting cells, including macrophages and DCs (13) (Figure 1). PD-L1 is also expressed on non-hematopoietic cells (solid-tumor, endothelial, and epithelial cells) and consequently helps in protecting tissues against immune-mediated injury (14, 15).

PD-1-PD-L1/PD-L2 ligation inhibits Th1 cytokine secretion, T cell proliferation (thus promoting T-cell apoptosis), and cytotoxic T lymphocytes (CTL)-mediated killing. This pathway is fundamental in the physiologic setting, preserving the immunologic balance after the initial T-cell response, which prevents collateral tissue damage, overactivation, and the irregular increase in autoreactive T cells (16). In presence of malignancy, the upregulation of the PD-1/PD-L1 pathway prevents tumor-reactive T cells to be activated and functioning, thus fostering immune escape and tumor growth (17, 18).

For these reasons, the potential benefit of antibody blockade of the PD-1/PD-L1 pathway has been evaluated in patients affected by solid tumors such as renal cancer, melanoma, non-small cell lung cancer, and hematologic malignancies (e.g., Hodgkin Lymphoma and MM).

Preclinical studies showed a higher expression of PD-L1 on MM patients' plasma cells rather than on plasma cells isolated from patients with monoclonal gammopathy of undetermined significance (MGUS) or on normal plasma cells (19). Rosenblatt et al. detected the PD-1 expression on circulating T cells in progressive MM patients, whereas the PD-1 expression on T-cells was reduced in patients with response after high-dose chemotherapy. They also examined PD-1 inhibition on *ex vivo* T-cell response to DC/tumor fusions (“a cancer vaccine in which autologous tumor was fused with dendritic cells, resulting in the presentation of tumor antigens in the context of DC-mediated costimulation”). By using an anti-PD-1 antibody, they promoted the polarization of T cells toward an activated phenotype that expressed Th1 compared with Th2 cytokines and the reduction and the killing of regulatory T cells (16, 20). As a consequence, the PD-1/T cells binding causes anergy (mainly through a blockade of B7-H1 [B7 homolog 1 protein]-PD-1 interaction) and apoptosis (through the inhibition of the anti-apoptotic gene bcl-xL and the activation of the proapoptotic gene Bim) (21, 22).

Moreover, PD-L1 is also expressed on the bone marrow microenvironment accessory cells, such as plasmacytoid DCs and MDSCs. In *in vitro* experiments, PD-1 inhibition restored the ability of plasmacytoid DCs to generate CTL killing of myeloma targets (23–25). PD-L1 on MDSCs may synergize with tumor cells to induce tolerance; therefore, its blockade may contribute to the inhibition of MM cell growth. Finally, PD-1 expression is increased on MM patient-derived NK cells, with an associated loss of effector cell function, which can be subsequently restored by the PD-1 blockade (26).

### PD-1/PD-L1 inhibitors in multiple myeloma: preclinical data and synergism with other compounds and strategies

PD-1 blockade alone is clinically most effective in tumors (e.g., melanoma and lymphoproliferative diseases) that show high levels of infiltrating effector cells in the tumor background and a high mutational burden, which can result in the production of neo-antigens and non-self epitopes hit by high-affinity T cells. Conversely, MM presents a limited neo-antigen profile, with a less intense infiltration of effector cells and a lower mutational activity than in solid tumors (27). In fact, MM pre-clinical studies showed that checkpoint blockade efficacy could be improved if associated with treatments able to intensify the activity of myeloma-reactive T cells, such as transplantation, cellular therapies, anti-CD38 antibodies, chimeric antigen receptor (CAR) T cells, and IMiDs.

IMiDs enhance T-cell responsiveness to antigen-presenting cells (APC), polarize T cells toward a Th1 phenotype, inhibit MDSC and Tregs, and downregulate PD-L1 expression on tumor cells (28–30). In particular, lenalidomide promotes apoptosis in cancer cells and stimulates NK and T cells, favoring NK-mediated tumor detection and killing (31).

In a preclinical study, NK cells and T cells were sorted by fluorescence-activated cell sorting (FACS) and then separately co-cultured with CD138^+^ MM cells from relapsed and/or refractory MM (RRMM) patients, plus anti-PD-1, anti-PD-L1, together or alone, and in association with lenalidomide. As a consequence, Görgün et al. demonstrated that the anti-myeloma toxicity deriving from the effector cells is enhanced by the PD-1/PD-L1 inhibition. Compared to T cells, NK cells showed a higher cytotoxicity. Moreover, the cytotoxicity induced by lenalidomide was further increased by checkpoint blockade (30). In another study, isolated CD4^+^/CD8^+^ T cells and NK cells from patients with MM were co-cultivated with autologous plasmacytoid DCs, together with the anti-PD-L1. In this way, Ray et al. proved that the use of anti-PD-L1 activated more deeply CD8^+^ T- and NK-cell cytotoxicity rather than CD4^+^ T-cell mediated killing (24).

Promising clinical results observed with IMiDs and anti-PD-1 combinations encouraged subsequent studies with agents that induce immune activation in the tumor microenvironment while stimulating myeloma cell killing. The anti-CD38 daratumumab kills malignant PCs through traditional antibody-dependent cellular cytotoxic mechanisms that are potentially able to control myeloma disease. In responding patients, daratumumab depletes subpopulations of Tregs and MDSCs in the myeloma microenvironment, stimulates T-cell expansion and increases T-cell clonality (32). These findings constituted the rationale for daratumumab associated with PD-1/PD-L1 blockade with or without IMiDs (NCT01592370, NCT03000452, and NCT02431208).

The anti-SLAMF7 monoclonal antibody elotuzumab has a dual mechanism of action that directly activates NK cells and causes the induction of NK cell-mediated antibody-dependent cellular cytotoxicity. A study on a mouse tumor model showed that the efficacy of elotuzumab was significantly higher when coadministered with anti-PD-1 antibody, thus promoting tumor-infiltrating NK and CD8^+^ T-cell activation, as well as augmented intratumoral cytokine and chemokine release. These data provided the rationale for the evaluation of elotuzumab/anti-PD-1 combination in MM patients (33).

It has been shown that cytotoxic therapy depletes suppressor populations and favors the reactivation of myeloma immunity. In a murine model, PD-L1 inhibition was given after stem-cell transplantation and cell vaccination administration, improving the survival of myeloma-bearing mouse models from 0 to 40% (34). One study showed that lymphopoietic reconstitution after stem-cell transplantation resulted in the depletion of regulatory T cells and the concomitant expansion of some MM clones. The inhibition of PD-1 significantly enhanced the proliferation and cytokine production of CD8^+^CD28^neg^PD-1^+^ T cells. Nivolumab treatment also increased the secretion of the cytokines IFNγ, IL2, and TNFα. These results suggested that checkpoint blockade can potentially improve or restore T-cell responses in this patient population (35). This provides the rationale to study this drug as maintenance in the post-transplant setting.

In the context of MM, the efficacy of PD-1/PD-L1 blockade may also be favored by the use of tumor vaccines, which can be administered for the expansion of MM-reactive T-cell clones and, as a consequence, for the activation with checkpoint blockade (20, 36).

Very recently, DC vaccination associated with PD-1 blockade and lenalidomide was investigated by Vo et al. in a myeloma-bearing mouse model. This combination inhibited myeloma tumor growth more effectively than other groups of agents, reducing immune suppressor cells (such as MDSCs, M2 macrophages, and Tregs), increasing immune effector cells, and enhancing the activity of NK cells and CTLs. This established a strong two-way anti-myeloma immunity through the inhibition of immunosuppressive cells and the activation of effector cells (37).

Interestingly, the combination of a PD-1 antibody with a CAR T cell showed an improved efficacy, even if the overexcitation of immune effectors could result in potential toxicity. In the study by Cherkassky et al. (38), the effector function of CD28 CAR T cells in a pleural mesothelioma-bearing orthotopic murine model was restored by the use of PD-1 antibody checkpoint blockade. These results allowed an improved understanding of the exhaustion of human CAR T-cell in solid tumors, suggesting that the effectiveness of CAR T-cell therapies may be improved by PD-1/PD-L1 blockade also in the context of hematological malignancies (38). Further studies are needed for the evaluation of the potential synergism of CAR-T therapies and anti-PD-1/PD-L1 checkpoint inhibitors in MM.

PD-1 blockade may also be effective when combined with radiotherapy, resulting in epitope spreading and increased antigen presentation by local APC (39). Temporal PD-L1 upregulation in the irradiated tumor suggested intrinsic mechanisms that inhibit immune responses after radiotherapy, and provided the rationale for blockade of PD-L1 combined with radiotherapy to overcome these mechanisms (40).

## PD-1/PD-L1 monoclonal antibodies: updated clinical results and safety considerations

MAbs targeting both PD-1 (pembrolizumab and nivolumab) and PD-L1 (durvalumab) have been evaluated for MM treatment.

Nivolumab is a human IgG4 mAb that blocks the interaction with PD-L1 and PD-L2 by binding to the PD-1 receptor on activated immune cells (13). Nivolumab has a very high binding affinity to PD-1 with about 80% of saturation reached in < 1 day following a single nivolumab infusion at 3 mg/kg; PD-1 occupancy is higher than 70% for almost 60 days, with detectable levels of PD-1 receptor occupancy for more than 3 months (41). Nivolumab clearance is not affected by renal or hepatic impairment (42).

Nivolumab as single agent did not show objective responses in a phase Ib trial enrolling 27 RRMM patients (43). The reasons for the lack of effectiveness in MM are unclear, but they may be related to the immunosuppressive nature of the microenvironment. To be effective, immune checkpoint therapy requires T cells to be able of being activated and, consequently, to have an exhausted phenotype instead of an anergic or senescent one (the key for therapeutic response is considered the reversal of exhausted T cells, rather than the genesis of new ones). In clinical studies on MM, clonal cytotoxic CD8^+^ T cells are the only T cells that showed to have an impact on survival; however, they did not show the exhausted phenotype. Rather, their phenotype (CD8^+^TCRVβ^+^CD57^+^CD28^−^) suggested the presence of terminally differentiated, antigen-specific, senescent cells that were no more able to proliferate after stimulation. Besides, in contrast with solid tumors and tumor-infiltrating lymphocytes, the low expression of PD-1 on clonal bone marrow cytotoxic T cells suggested that, in MM, the local immuno-suppressive mechanisms involving PD-1/PD-L1 interactions are less active (44).

Combinations of nivolumab with pomalidomide-dexamethasone (Pd) and other mAbs, such as daratumumab (NCT01592370) and elotuzumab (NCT02726581), have been designed in more recent trials, but data are still not available.

Durvalumab is a human IgG1k antibody targeting PD-L1. Weight-based durvalumab dose (10 mg/kg every 2 weeks) and fixed durvalumab dose (1,500 mg every 4 weeks or 750 mg every 2 weeks) demonstrated similar PK features, with patient and disease characteristics that did not affect drug bioavailability (45). In the MM setting, no clinical data on durvalumab are available and phase I studies investigating durvalumab plus IMiDs are currently on clinical hold on the basis of the results of the KEYNOTE-183 and KEYNOTE-185 trials, which will be described below.

Pembrolizumab is an IgG4k humanized anti-PD-1 mAb. Neither pharmacokinetics nor renal/hepatic impairment are affected by age, thus dose adjustments are not needed (46).

In MM, no data are available on pembrolizumab as single agent. In a phase I study including RRMM patients, pembrolizumab (maximum tolerated dose: 200 mg every 21 days) associated with lenalidomide-dexamethasone (Rd) showed a partial response (PR) rate of 50%. Any-grade treatment-related AEs occurred in 48 (94%) patients, albeit grade ≥3 AEs were observed in 33 (65%) patients. Grade 3 irAEs included increase in transaminases (2%), and renal failure (2%).

Pembrolizumab (200 mg every 2 weeks) was also combined with Pd, showing a PR rate of 60% (47, 48). Thirty-five (73%) patients experienced any-grade treatment-related AEs, albeit ≥3 AEs were observed in 20 (42%) patients. Grade 3–4 irAEs included hypothyroidism (4%), adrenal insufficiency (2%), hepatitis (2%), and pneumonitis (2%).

Based on these studies, two randomized phase-III trials were designed. In the KEYNOTE-185 trial (NCT02579863), pembrolizumab-Rd vs. Rd alone was investigated in transplant-ineligible NDMM patients. On the 3^rd^ of July, 2017, after that interim data had been presented to the Data Monitoring Committee (DMC), the FDA put a hold on the trial because of an increase in deaths in the pembrolizumab arm. Three hundred and one of the planned 640 patients were enrolled (median age 74 years). After a median follow-up of 6.4 vs. 6.9 months, there were 19 (13%) deaths in the pembrolizumab-Rd arm (6 from PD, 13 from AEs) vs. 9 (6%) patients in the Rd arm (1 from PD, 8 from AEs); 6 (4%) treatment-related deaths were observed; 4 (3%) were related to pembrolizumab (1 cardiac arrest, 1 pneumonia; 1 myocarditis, 1 cardiac failure). The other AEs that led to death were: cardiorespiratory arrest and pulmonary embolism (2 patients each), intestinal ischemia, large intestinal perforation, sudden death, suicide, and sepsis (1 patient each). In the Rd arm, the AEs that led to death were myocardial infarction and sudden death (2 patients each), acute cardiac failure, upper intestinal hemorrhage, respiratory failure (1 patient each). This translated into an increased risk of death with pembrolizumab (HR for OS: 2.06; 95% CI 0.93–4.55; *P* = 0.97). The rates of severe (grade 3–5) toxicities were 72% in the experimental arm vs. 50% in the control arm. The rates of serious AEs (SAEs) were 54 vs. 39%, respectively. The rates of discontinuation for AEs were 21 vs. 8%, respectively. AEs (all grades) with more than 5% of difference between arms included: constipation, pyrexia, vomiting, rash, hypothyroidism, oral candidiasis, hyperthyroidism, pruritus, pneumonia, and decrease appetite. In the pembrolizumab-Rd arm, irAEs reported in ≥2% of patients included: hypothyroidism (7%), hyperthyroidism (6%), colitis (2%), and skin reactions (13%). Median progression-free survival (PFS; HR 1.22; 95% CI 0.67–2.22, *P* = 0.75) was not reached in neither arm (49).

The second trial, KEYNOTE-183 (NCT02576977), evaluated pembrolizumab-Pd vs. Pd alone in RRMM patients who received ≥2 lines of treatment including an IMiD and a proteasome inhibitor (PI). Similarly to what happened with KEYNOTE-185, the FDA halted the trial on the 3rd of July, 2017 on the basis of interim data provided to the DMC. The study enrolled 249 of the planned 300 patients (median age: 65 vs. 67 years in pembrolizumab-PD vs. PD arms, respectively, median duration of therapy 4.4 cycles). After a median follow-up of 7.8 vs. 8.6 months, 29 (23%) vs. 21 (17%) patients died (16 from PD, 13 from AEs vs. 18 from PD, 3 from AEs). In the pembrolizumab-Pd arm, 4 (3%) treatment-related deaths occurred: 2 (1.5%) were related to pembrolizumab (1 myocarditis, 1 Steven-Johnson syndrome [SJS]); 1 patient died of neutropenic sepsis. The other AEs that led to death were sepsis (3 patients), pericardial hemorrhage, myocardial infarction, cardiac failure, and respiratory tract infection (1 patient each). In the Pd arm, the AEs that led to death were pneumonia and anemia (1 patient each). The median OS was not reached vs. 15.2 months (HR, 1.61, 95% CI, 0.91–2.85; *P* = 0.95) in the pembrolizumab-Pd vs. Pd arm. The rate of grade 3–4 AEs was 75% in the experimental arm vs. 63% in the control arm. The rates of SAEs were 63 vs. 46%, respectively, 20 vs. 8% discontinued for AEs. AEs (all grades) occurred in ≥20% of patients were: neutropenia, anemia, fatigue, constipation, pyrexia, pneumonia, and thrombocytopenia. No SAEs had more than 5% of difference between arms. In the pembrolizumab-Pd arm, irAEs included: skin reaction (5%), pneumonitis (4%), hyperthyroidism (3%), infusion reaction and myopathy (2% each), SJS, myocarditis, hepatitis, and iridocyclitis (1% each). Median PFS was similar between the two arms (5.6 vs. 8.4 months; HR 1.53, 95% CI 1.05–2.22; *P* = 0.98) (50).

Both trials determined that the risk-benefit profile of adding pembrolizumab to Rd or Pd was unfavorable.

### Immune-related AEs

The precise pathophysiology of irAEs is unknown, although likely related to the ability of immune checkpoints of preserving the normal immunologic homeostasis. These irAEs generally develop within a few weeks or months from the start of treatment, but they may occur at any time, including after stopping therapy (9). Although every organ system may be affected, irAEs usually involve skin, gastrointestinal tract, liver, and endocrine glands (51). Causes of severe irAEs remain unclear. One of the hypotheses was prompted by the association with underline germline genetic factors, since genes may influence the risk of specific autoimmune disorders. Another hypothesis was the association with the patient microbiota (9). It is important to promptly identify the occurrence of irAEs. Most of the times, the diagnosis and the treatment are based on patient-related symptoms, but sometimes blood test and/or imaging can be helpful (e.g., for hepatitis, colitis, or pneumonia). The optimal management is based on clinical experience, mainly in the treatment of solid tumors, since no prospective trials are available. The backbone of irAE therapy is immunosuppression with corticosteroids, with the addition of other immunosuppressive agents if there is no rapid improvement. Most of AEs promptly resolve, and available data do not show a negative impact of immunosuppression on the effectiveness of checkpoint inhibitors (52).

AEs provided evidence of activation of the patient immune system, but irAEs are not required for efficacy. Data from the literature on the correlation between the occurrence of irAEs and treatment efficacy are controversial (9). A *post-hoc* analysis of KEYNOTE-183 and KEYNOTE-185 was performed to examine the correlation between irAEs and efficacy. In the KEYNOTE-185 study, 68 vs. 44% of patients in the pembrolizumab-Rd vs. Rd arms had an irAE (grade ≥3: 36 vs. 8%). Despite the overall higher rate of irAEs in the pembrolizumab-Rd arm, the overall response rate (ORR) was similar to the one in the Rd arm (64 vs. 62%). Nevertheless, in the pembrolizumab-Rd arm, ORR was higher in patients who experienced an irAE, as compared to patients who did not (73 vs. 45%). Similarly, in the Rd arm, the ORR was 73% in patients with an irAE vs. 53% in those without (53). These results might suggest that anti PD-1 are more effective in patients with activation of the immune system (evidence of which can be considered the development of irAEs). The KEYNOTE-183 study enrolled RRMM patients, who typically have a less effective immune-system as compared to NDMM patients. In this study, both the rate of irAEs and the differences between arms (58 vs. 45% of patients in the pembrolizumab-Pd vs. Pd arms; grade ≥3: 18 vs. 13%) were lower if compared to the NDMM setting (KEYNOTE-185). In the pembrolizumab-Pd arm, the ORR was 37% in patients who developed an irAE, not significantly different than the rate in those without an irAE (31%). In the Pd arm, a trend was noted for improved ORR (49%) in patients who experienced an irAE, as compared to 33% in those who did not. Altogether, these results suggest a higher risk of irAEs in NDMM patients, who probably have a more effective immune system as compared to heavily pretreated patients. A higher effectiveness of PD-1 inhibitors in patients with irAEs still needs to be demonstrated.

## Conclusion

During the last decade, therapeutic strategies in MM patients have vastly improved thanks to the introduction of mAbs in association with backbone regimens. Based on pre-clinical data, the PD-1/PD-L1 axis may be a good target for mAbs, allowing immune cells to detect and kill neoplastic cells. However, the outcomes of checkpoint blockade alone in MM are inferior to the ones obtained in solid tumors, most likely due to the reduced immune function typical of the immune system of patients affected by MM. In phase II trials, potentially better results have been observed in association with IMiDs, probably due to the possible synergistic effect on the immune system. Nevertheless, despite these promising preliminary data, the toxicity reported in two randomized phase III trials with pembrolizumab associated with lenalidomide and pomalidomide led the FDA to halt trials exploring these combinations. The safety concerns are related to the mechanism of action of both drug classes, as they modify the behavior of immune cells. Moreover, patients treated with mAbs receive continuous therapy with steroids, either as part of treatment or to reduce irAEs. This leads to immunosuppression and may increase the risk of infections, which can ultimately cause drug discontinuation and reduce the efficacy of the treatment itself.

The effective possibility to modulate the immune system would be a great advancement. However, there are still many open issues. We need to ponder how to select and monitor patients for this typology of treatment, and to determine the best and safest drug combination as well as the most suitable time point of administration during the disease course. Moreover, the detection of biomarkers that can potentially predict responses and/or toxicities might help clinicians balance efficacy with safety.

To conclude, more mature safety data and a deeper analysis of the biologic mechanisms will be essential to understand if PD-1/PD-L1 inhibitors may be included in the armamentarium for the treatment of MM patients.

## Author contributions

SO, RT, MD, MB, and FG conceptualization and methodology. SO, RT, MD, and FG writing—original draft. SO, MB, and FG supervision. SO, RT, MD, MB, and FG writing—review and editing.

### Conflict of interest statement

SO has received honoraria from Takeda, Amgen, and Celgene. FG has received honoraria from Takeda, Amgen, Celgene, Janssen, and BMS; has served on the advisory boards for Takeda, Seattle Genetics, Celgene, and Roche. MB has received honoraria from Sanofi, Celgene, Amgen, Janssen, Novartis, AbbVie, BMS, and research funding from Celgene, Janssen, Amgen, BMS, Mundipharma, Novartis, and Sanofi. The remaining authors declare that the research was conducted in the absence of any commercial or financial relationships that could be construed as a potential conflict of interest.

## Abbreviations

MM: multiple myeloma
NK: natural killer
DC: dendritic cells
MDSC, myeloid-derived suppressor cells, TCR: T cell receptor
MHC-Ag: major histocompatibility complex-antigen
PD-1: programmed cell death 1
PDL-1: programmed cell death ligand 1.
